# Contemporary considerations in the management and treatment of lower pole stones

**DOI:** 10.1590/S1677-5538.IBJU.2021.0010

**Published:** 2021-02-28

**Authors:** Ridwan Alam, Brian R. Matlaga, Ayman Alam, Jared S. Winoker

**Affiliations:** 1 Johns Hopkins University School of Medicine James Buchanan Brady Urological Institute Department of Urology Baltimore USA Department of Urology, James Buchanan Brady Urological Institute, Johns Hopkins University School of Medicine, Baltimore, USA

**Keywords:** Calculi, Therapeutics, Review Literature as Topic

## Abstract

The presence of lower pole stones poses a unique challenge due to the anatomical considerations involved in their management and treatment. Considerable research has been performed to determine the optimal strategy when faced with this highly relevant clinical scenario. Standard options for management include observation, shock wave lithotripsy, retrograde intrarenal surgery, or percutaneous nephrolithotomy. Indeed, each approach confers a distinct set of risks and benefits, which must be placed into the context of patient preference and expected outcomes. The current state of practice reflects a combination of lessons learned from managing calculi not only in the lower pole, but also from other locations within the kidney as well.

## INTRODUCTION

The management of urolithiasis has gained significant interest over the past two decades, perhaps as a result of the increased prevalence worldwide (1–3). Children, too, are presenting with stone disease at higher rates than in previous years (4). The magnitude of this condition is only amplified when considering that these patients suffer from a lifetime recurrence risk of up to 50% (5). As such, the cumulative economic burden of urolithiasis is large and increasing rapidly. In the United States alone, the annual expenditure to care for these patients was estimated at $2.1 billion in 2000 and is projected to increase by an additional $1.24 billion per year by 2030 (6). Therefore, considerable effort has been devoted to determine the most appropriate management strategy for patients suffering from urolithiasis, with a particular focus on stone-free rates.

Stones within the kidney are most likely to develop in the lower pole, accounting for approximately 35% of cases (7). Removal of kidney stones is typically achieved via one of three methods: shock wave lithotripsy (SWL), retrograde intrarenal surgery (RIRS), and percutaneous nephrolithotomy (PCNL). Each intervention possesses unique merits and challenges based on stone characteristics and anatomical considerations. Lower pole stones pose a particularly unique challenge given the relative difficulty of clearing calculi from this space, even after adequate fragmentation. Indeed, both the American Urological Association (AUA) and European Association of Urology (EAU) have published guideline recommendations for the management of lower pole stones (8–10). However, these guidelines differ slightly due to the lack of large randomized controlled trials and high quality data on this topic.

Nevertheless, there are a number of studies that have been performed over the years to elicit an understanding of how best to achieve stone-free status for lower pole stones. Still, there is a large research gap which precludes analysis of other treatment-related outcomes such as postoperative quality of life and resource utilization. We describe the current practice of lower pole calculi management and review the data for each treatment strategy.

### 

#### Surveillance

The increased utilization of axial imaging has resulted in a concomitant rise in the incidental detection of asymptomatic kidney stones. The prevalence of asymptomatic urolithiasis has been estimated at 8%, with a mean size ranging from 3 to 10mm (11–13). Approximately 25-50% of these are found in the lower pole, where it is believed that calculi are less likely to resolve spontaneously due to its dependent location in the kidney relative to the ureteropelvic junction. Despite this, the majority of lower pole stones remain asymptomatic (12, 13). There is, however, considerable debate regarding the need for intervention in this patient cohort due to the limited number of high quality studies on the natural history of lower pole calculi. As such, there is no uniform consensus with regards to the need for monitoring or intervention in patients with asymptomatic lower pole stones.

Recognizing these shortcomings, the AUA allows for the active surveillance of asymptomatic, non-obstructing stones with only a low level of confidence (9). No specific surveillance protocol is defined and the decision to pursue intervention is largely based on shared decision-making between the clinician and patient. Similarly, the EAU also allows for observation and cites the weak level of evidence available on this topic (10). Annual follow-up is suggested to monitor the stones, with clinicians advised to consider intervention for asymptomatic stones demonstrating growth.

The reporting on surveillance varies widely, thus contributing to the difficulty in managing asymptomatic lower pole stones. In a retrospective review of 300 men at the Minneapolis Veterans Affairs Medical Center, 168 (56%) were found to have lower pole stones (13). Over the follow-up period, these lower pole stones were found to be more likely to grow compared to their non-lower pole counterparts (61% vs. 47%, P=0.002). However, there were no notable differences in the proportions of patients experiencing pain (40%) or requiring intervention (20%) between the lower pole and non-lower pole groups. A separate study from the group at Dartmouth found slightly differing results in their cohort of 160 patients: while there was no difference in the rate of intervention (19% for lower pole vs. 20% for non-lower pole, P=0.83), patients with non-lower pole stones were more likely to become symptomatic than patients with lower pole stones (41% vs. 24%, P=0.05) (12). Unlike in the prior study, no significant difference in growth was detected between the two groups (19% vs. 19%, P >0.99). Importantly, non-lower pole stones were much more likely to pass spontaneously compared to lower pole stones (15% vs. 3%, P=0.02). A contemporary study involving 293 patients from China found that lower pole stones were less likely to be symptomatic (HR 0.24, P <0.001) and less likely to grow (HR 0.35, P=0.02), but also less likely to pass spontaneously (HR 0.29, P <0.001) when compared to stones located in other parts of the kidney (14). Similar to prior studies, intervention rates did not differ based on the calyceal location of the stone (HR 1.03, P=0.95).

There is one randomized trial which provides credence to the idea that surveillance is a reasonable option in asymptomatic lower pole stones. In this 2010 study, 94 patients with asymptomatic lower pole stones ≤20mm were prospectively randomized to PCNL (n=31), SWL (n=31), and observation (n=32) (15). Post-procedural stone-free rates were 100% and 61% for the PCNL and SWL groups, respectively, at 12 months. At the same timepoint, only 1 patient (3%) in the observation group had experienced spontaneous passage. While symptom occurrence was not explicitly stated, only 7 of the 32 patients (19%) in the observation group ultimately required intervention at a median 22.5 months after enrollment. Furthermore, renal scintigraphy demonstrated that none of the patients in the observation group experienced renal scarring at 12 months, whereas 3% and 16% of patients in the PCNL and SWL groups, respectively, did.

Synthesis of this data has proven challenging, as demonstrated in a 2010 systematic review of asymptomatic urolithiasis (16). Although the primary focus of this study was not lower pole stones, the authors concluded that surveillance of lower pole stones is a reasonable option if the stone burden is ≤10mm. This conclusion is derived from a single, small study of 24 patients with asymptomatic lower pole stones demonstrating that spontaneous passage was achieved in 50% of patients with stones <5mm, 16% with stones 5-10mm, and 0% with stones >10mm (17). However, the EAU has taken this same study to conclude that surveillance is most sensible for stones <5mm (10).

Ultimately, surveillance for lower pole stones is reasonable in the absence of symptoms such as pain, infection, and obstruction. While intervention may never be required, patients should be counseled on the possibility of acute symptom development due to the low likelihood of spontaneous passage. Patients who are unable to follow-up for monitoring or do not have regular access to immediate medical care (e.g., airline pilots, military servicepersons) may be best served with upfront intervention for their asymptomatic lower pole calculi as a prophylactic measure.

#### Shock wave lithotripsy

For patients who require intervention, SWL presents a unique opportunity for treatment with a palatable risk profile. This non-invasive option utilizes shock waves to fragment stones into smaller sizes, which may have a better chance of spontaneous passage. As there is no active extraction process involved with this procedure, stone-free rates are generally lower for SWL than for RIRS or PCNL (18, 19). There is mounting evidence to suggest that this trend is observed, and perhaps even amplified, when limited to lower pole stones (20, 21). This is because the residual fragments after SWL often remain in the lower pole, thereby resulting in recurrent stone formation. Given the time constraints of SWL, larger stones in the lower pole are more likely to result in larger residual fragments and necessitate repeat therapy. Therefore, while SWL is not contraindicated in the management of lower pole stones, the general consensus is that larger stones in the lower pole should be treated using alternative therapies.

Indeed, the AUA allows for the use of SWL when managing lower pole stones ≤10mm (9). However, the guidelines explicitly advise against offering SWL as first-line therapy for lower pole stones >10mm due to the significantly diminishing success of this modality when compared to RIRS or PCNL, especially when the stone burden exceeds 20mm. The EAU largely shares this opinion as well, noting the inverse relationship between stone-free rate and stone size when employing SWL (10). A small but notable difference is that SWL can be considered as a first-line option for stones up to 20mm. Nevertheless, certain factors have been identified which may impair the success of treatment by SWL, such as the presence of a steep infundibulopelvic angle, long calyx, long skin-to-stone distance, narrow infundibulum, and stone more resistant to shock wave therapy. In these cases, clinicians are advised to consider alternative treatments even if the stone burden is small.

The landmark study Lower Pole I examined 128 patients with lower pole stones who were randomly assigned to SWL (n=68) or PCNL (n=60) (22). Treatment failure, which was defined as the need for a secondary procedure, occurred in 9 patients (13%) who underwent SWL and in none of those who underwent PCNL. Stone-free rates at 3 months demonstrated an even greater disparity, with 37% in the SWL group becoming stone-free compared to 95% in the PCNL group (P <0.001). The difference in stone-free rate widened between SWL and PCNL as the stone size increased, with PCNL consistently performing better. In fact, a stone-free rate of greater than 50% was achieved in the SWL group only if the stone burden was <10mm; beyond this threshold, stone-free status was achieved in less than a quarter of patients. In 38 SWL patients with anatomical data, the presence of a steep infundibulopelvic angle, long calyx, or narrow infundibulum were not found to be significant predictors of stone-free status. While PCNL was overwhelmingly more successful than SWL in treatment of lower pole stones, it did come at the expense of increased hospitalization (2.7 days vs. 0.6 days, P <0.001) and a trend toward increased complications (22% vs. 11%, P=0.09). Finally, treatment of the stone was associated with an increased quality of life, as measured by a survey at 3 months, but there was no significant difference between the SWL and PCNL groups. As a result of this study, it was suggested that SWL should be reserved for patients with a lower pole stone burden of ≤10mm.

Using the lessons from the Lower Pole I study, Pearle et al. randomized 67 patients with lower pole stones measuring ≤10mm into treatment by SWL (n=32) or RIRS (n=35) (23). There were 5 treatment failures in both groups. Three-month stone-free rates were not found to be statistically different between the two groups (35% ESWL vs. 50% RIRS, P=0.92). All patients were discharged home the same day, but SWL patients were able to return to baseline activities much quicker than RIRS patients. Furthermore, SWL patients required fewer pain medications postoperatively than RIRS patients (5.6 pills vs. 14.7 pills, P=0.02) and were more likely to choose to undergo the same procedure again (90% vs. 63%, P=0.03). While there was no difference in the rate of postoperative complications (23% SWL vs. 21% RIRS, P=0.84), SWL trended toward a lower rate of intraoperative complications (3% vs. 20%, P=0.06). In addition, the operative time for SWL was significantly shorter than that for RIRS (65.5 minutes vs. 90.4 minutes, P=0.01). This study, therefore, supported the use of either SWL or RIRS in the management of lower pole stones measuring ≤10mm, with the added caveat that SWL was associated with increased patient satisfaction and a shorter time to recovery.

In a contemporary summary of the data, Donaldson et al. performed a systematic review and meta-analysis of patients with lower pole stones to provide level 1a evidence regarding the comparative effectiveness of SWL, RIRS, and PCNL (21). Only randomized trials were included in this study, including the two mentioned above. Two studies compared SWL to PCNL and five compared SWL to RIRS. In brief, the stone-free rate at 3 months favored PCNL over SWL (RR 2.04, P <0.001) and RIRS over SWL (RR 1.31, P=0.007). While these relationships were maintained over the entire size spectrum, the magnitude of benefit dropped considerably for stones ≤10mm. These findings largely establish the rationale for limiting SWL to patients with lower pole stones ≤10mm.

Despite the findings from the Lower Pole I study, which demonstrated no association between SWL success and anatomic factors, it is rather universally accepted that denser stones and increased skin-to-stone distance portend a worse prognosis. Although the referenced studies did not exclusively examine lower pole stones, they found that a skin-to-stone distance of >9cm or a stone attenuation of >10.000 Hounsfield units on computed tomography were associated with a lower likelihood of success using SWL (24, 25). Therefore, therapies other than SWL should be considered if unfavorable factors are involved, even if the lower pole stone burden is ≤10mm.

SWL for lower pole stones offers an attractive, non-invasive treatment option for individuals wishing to minimize the risks of surgery. There are, however, several considerations when employing SWL, including stone characteristics and anatomic factors. In this regard, patient selection is crucial to optimize postoperative outcomes and reduce the need for repeat procedures. While SWL is an important and useful option in the treatment of lower pole urolithiasis, it is rather universally accepted that larger calculi, particularly those >10mm, should not be treated using this modality as a first-line option.

#### Retrograde intrarenal surgery

With improvements in fiberoptic and laser technology, RIRS has gained popularity among both patients and providers due to its minimally invasive approach and perceived ease of use. In fact, a survey of chief residents and recent residency graduates demonstrated that 87% of respondents felt very comfortable with ureteroscopy compared to 72% for SWL and 48% for PCNL (26). Despite this, stone-free rate after RIRS is estimated to be only 60% for stones located anywhere in the kidney (27). As in the case of SWL, there is an inverse relationship between stone burden and stone-free rate for RIRS (19). Furthermore, the challenges encountered in the management of lower pole stones are similar between SWL and RIRS (28). As such, recommendations for the management of lower pole stones with RIRS almost mirrors that for SWL.

For example, the AUA recommends the use of either SWL or RIRS for lower pole stones ≤10mm (9). Unlike in SWL, however, there is no specific guideline statement against the use of RIRS as a first-line therapy for stones >10mm. In fact, RIRS appears to be the most versatile surgical option for lower pole stones, as there are no strict cutoff parameters that restrict its use on either the high or low end of the size spectrum. However, the EAU takes a slightly different stance on this issue. While RIRS is allowed, and even subtly encouraged over SWL, for lower pole stones ≤20mm, PCNL is clearly listed as the preferred first-line option for stones >20mm (10).

At first glance, it appears that RIRS may be less desirable than SWL for the management of lower pole stones. Indeed, the first randomized trial comparing SWL to RIRS, published in 2008, found no difference in stone-free rates, but RIRS was associated with lower patient satisfaction and a longer convalescence period (23). Importantly, all patients in this study had lower pole stones ≤10mm. Since then, four additional trials have demonstrated that RIRS does in fact confer a benefit with respect to stone-free rates, but these benefits are more apparent in stones measuring >10mm (29–32). In the 2015 meta-analysis, the risk ratio of achieving stone-free status was 1.50 in favor of RIRS over SWL if the stone measured between 10 and 20mm (P <0.001) (21). However, this dropped to 1.11, with RIRS still favored over SWL, if the stone measured <10mm (P=0.004). Furthermore, the study by Singh et al. demonstrated findings contradictory to Pearle et al. on almost every account of patient quality of life outcomes - higher satisfaction (2.82 vs. 2.17, P=0.03) and higher willingness to undergo the same procedure (84% vs. 50%, P=0.002) were reported in the RIRS group when compared to the SWL group (32). Perhaps, then, it is unsurprising that the jury is still out regarding the superiority of SWL over RIRS, or vice versa, and therefore finds the use of RIRS reasonable in all instances when SWL could be employed.

With respect to RIRS versus PCNL, however, there is only one randomized trial examining the effectiveness of these procedures in lower pole stones. Published only as an abstract, the findings from the Lower Pole II study demonstrated that there was no significant difference in stone-free rates among stones measuring >10mm (46% vs. 67%, P=0.29) (33). Unsurprisingly, PCNL was associated with a longer hospital stay (2.8 days vs. 0 days, P <0.001) and recovery time (23.5 days vs 10.0 days, P <0.05) than RIRS. However, this was a very small study with only 28 patients (13 in RIRS, 15 in PCNL) that was published when RIRS was in its infancy. Therefore, aside from drawing intrigue as the only randomized trial in this space, this study carries very little clinical value in modern practice.

As such, conclusions about the utility of RIRS versus PCNL in the management of lower pole calculi are derived from the body of literature examining kidney stones, regardless of calyceal location. In a systematic review and meta-analysis comparing RIRS to PCNL in the treatment of kidney stones, De et al. reviewed 8 non-randomized and 2 randomized studies (34). They found that patients who underwent PCNL had nearly 2.2 times greater odds of becoming stone free when compared to patients who underwent RIRS (P <0.001). However, it is unclear whether this difference varied with stone size, as this was not analyzed. Furthermore, PCNL was associated with higher complication rates (OR 1.61, P=0.01) and longer hospitalizations (weighted mean difference [WMD]+1.3 days, P <0.001).

As a standalone procedure, RIRS demonstrates acceptable performance when evaluating stone clearance. However, as in the case of SWL, stone burden is an important predictor of success. In a study of 90 patients with lower pole stones, those with a stone burden of ≤10mm, 10-20mm, and >20mm demonstrated three-month stone-free rates of 82%, 72%, and 65%, respectively, after RIRS (35).

Furthermore, the presence of a steep infundibulopelvic angle, long calyx, or narrow infundibulum were associated with treatment failure. Unsurprisingly, larger stones were associated with longer operative times. These results, however, are challenged by a contemporary study of patients with lower pole stones >20mm. In this retrospective review of 109 patients who underwent RIRS (n=32) or PCNL (n=77), there was no significant difference in the one-month stone-free rate (91% RIRS vs. 96% PCNL, P=0.26) (36). Furthermore, the operative times were similar between the two groups (67.5 minutes in RIRS vs. 62.5 minutes in PCNL, P=0.67). Taken in context, this study suggests that the success of RIRS is highly operator-dependent and that lower pole stones >20mm can be effectively managed using RIRS in experienced hands.

For the general population, the indications for RIRS largely mirror that of SWL. Although more involved than SWL, RIRS adequately fills a niche for small to medium lower pole stones, particularly those measuring 10-20mm, to achieve acceptable stone-free rates using a less invasive approach than PCNL. With its familiarity and versatility, RIRS is sure to remain a mainstay in the treatment of lower pole calculi.

#### Percutaneous nephrolithotomy

There are, of course, situations which necessitate the employment of more aggressive interventions to adequately treat patients with lower pole stones. Generally speaking, PCNL is favored in the treatment of larger calculi because its efficacy is less influenced by stone size than SWL or RIRS (37, 38). In fact, PCNL has almost entirely replaced the need for open or laparoscopic/robotic pyelolithotomy due to its high stone-free rate and more favorable risk profile (39, 40). While pyelolithotomy will continue to have a role in extremely limited situations, these aberrant cases are beyond the scope of standard practice and are perhaps best managed at a specialty center. Although PCNL carries a considerable learning curve to achieve excellence, the improved manipulation and visualization when compared to SWL and RIRS makes it an incredibly valuable tool in the management of lower pole stones (41–43).

To this end, the AUA appears to favor the use of PCNL in lower pole calculi >10mm but does not explicitly mandate its use over RIRS in the guideline statements (9). Instead, they insist that patients should be informed about the improved stone-free rate of PCNL at the expense of increased morbidity. On the other hand, the EAU very clearly recommends the use of PCNL for lower pole calculi >20mm and suggests that it should be highly considered for stones in the 10-20mm range as well (10).

The role of PCNL in the treatment of lower pole stones is firmly established. PCNL is considered the standard by which alternative therapies, such as SWL or RIRS, must seek to match using less invasive methods. The effectiveness of PCNL is without question - multiple studies have demonstrated that stone-free rates approach 100%, even among those with lower pole calculi (21, 34, 44). However, given the increased morbidity associated with PCNL, there has been an attempt to better define which alternatives can provide a more favorable risk profile without overly compromising treatment outcomes. Therefore, it is unsurprising that outcomes from the two randomized trials comparing PCNL to SWL for lower pole stones was greatly in favor of PCNL (RR 2.04, P <0.001) (21). The trend continues across the spectrum when stratifying the stones by size, but with varying magnitudes. When compared to their SWL counterparts, patients undergoing PCNL were 1.56 times more likely to become stone-free if their stone burden was ≤10mm (P=0.01), but this figure jumps to 4.02 if their stone burden was 10-20mm (P <0.001). As a result, the tradeoff to pursue SWL in an attempt to avoid the morbidity of PCNL was thought to be reasonable for lower pole stones ≤10mm.

As a result of the concern regarding the morbidity of PCNL, there has been a concerted effort to downscale the invasive nature of this operation by miniaturizing the PCNL. A litany of terms has been introduced to describe this approach, which we will refer to as the mini-PCNL. In brief, the mini-PCNL uses the same approach as conventional PCNL but with smaller instruments and access sheaths to minimize trauma to the kidney and surrounding tissues. Over time, mini-PCNL has demonstrated outcomes comparable to conventional PCNL but with lower morbidity (45). However, a meta-analysis of PCNL to RIRS for kidney stones, regardless of calyceal location, performed a subgroup analysis based on the use of conventional or miniaturized PCNL and found discrepant results (34). Compared to RIRS, conventional PCNL demonstrated higher stone-free rates (OR 4.32, P <0.001). On the other hand, RIRS demonstrated better stone-free rates than mini-PCNL (OR 1.70, P=0.03). Unfortunately, it is impossible to determine whether these outcomes may have been affected by stone location, and no studies to date compare the use of mini-PCNL to other therapies for stones exclusively located in the lower pole. Nevertheless, the indications for mini-PCNL are the same as those for conventional PCNL, and the utilization of either procedure remains as the discretion of the surgeon.

PCNL is a fantastic option for patients with lower pole calculi, especially if the stone burden is high. While it could theoretically be used to treat stones of any size, the increased risk of this procedure and ubiquitous availability of less invasive options means that PCNL is rarely employed for stones ≤10mm. Furthermore, PCNL requires a specific set of skills which can make the procedure technically challenging for clinicians who do not perform it on a regular basis. This likely reflects the AUA's decision not to explicitly recommend the use of PCNL for larger stones, as it allows for individuals who are more comfortable with RIRS to provide care for this population as well. However, as the data demonstrate, these patients may be best served by upfront PCNL if referral to a high-volume practitioner can be made in a timely manner.

#### Pediatric populations

With an increase in pediatric stone disease, consideration of this patient population becomes progressively more important. However, randomized studies on this topic are understandably very difficult due to the vulnerable nature of this population. This difficulty is compounded when attempting to study exclusively lower pole stones in pediatric patients. Therefore, there are no specific guidelines from the AUA or EAU on the management of lower pole stones for children. Instead, inferences are made based on retrospective observations from children treated for stones in other parts of the kidney as well as the lessons learned from the adult population.

As in the adult population, observation is generally favored in children with asymptomatic lower pole stones. A study from Turkey followed 242 children with asymptomatic lower pole stones measuring <10mm for a mean 3.4 years (46). Forty-two of these patients had asymptomatic lower pole stones in both kidneys at enrollment, resulting in a total of 284 stone occurrences. Over the follow-up period, 174 stones (61%) required intervention due to the development of pain, stone growth, obstruction, or infection. The mean time to intervention was 19.2 months. RIRS or mini-PCNL was used to treat 72 stones while the remaining 102 were treated by SWL. Stone-free rates were 82%, 79%, and 9% in the RIRS/mini-PCNL, SWL, and observation groups, respectively. The presence of anatomic renal anomalies, stones >7mm, or stones composed of cystine or struvite were associated with an increased odds of requiring intervention.

If treatment is indicated, the surgical options are the same for children as they are for adults. SWL is typically favored for its non-invasive approach but must be weighed against its lower stone-free rate. While the data are sparse, the stone-free rate for SWL hovers around 60-80% for lower pole stones with a mean size of 7mm (46, 47). The stone-free rate for RIRS improves to 75-85%, even though the stone size increased to a mean of 8-12mm (46, 48). When stratified by size, stones <15mm had a stone-free rate of 93% compared to 33% for those ≥15mm (P=0.01). There is no specific data for pediatric lower pole stones treated by PCNL, but if calyceal location is excluded, then PCNL demonstrates an even higher stone-free rate, ranging 70-90% (49–51). Importantly, the mean stone size was 20-23mm, thus demonstrating that this relatively invasive technique is usually reserved for only the largest of stones.

Despite the lack of strict guidelines, it appears that clinical management of lower pole stones in the pediatric population largely reflect that of the adult population, with very similar risk-benefit profiles. However, the long-term effects of childhood renal surgery are still unknown, so it would behoove clinicians to take a particularly careful approach with this patient population.

## DISCUSSION

The lower pole stone can be a challenging clinical entity. While stone size is the greatest driver of management decisions for calculi anywhere in the collecting system, anatomical considerations are further magnified in the lower pole. At the same time, other stone- and patient-related factors must be accounted for, all of which should be collectively evaluated on the foundation of shared decision-making between the physician and patient.

Organizational guidelines provide treatment recommendations based on maximum stone diameter or length of total stone burden in any single dimension (Table-1). This is not surprising because stone size has been repeatedly associated with outcomes of surgical success, such as stone-free rate and the need for secondary procedures. The inverse relationship between stone size and stone-free rate has been observed not only within all locations of the kidney, but also for each of the available surgical treatment options. Therefore, understanding the probability of surgical success balanced against the relative risks of a particular intervention, when stratified by stone burden and other pertinent factors, is paramount.

**Table 1:** Recommendations for the surgical management of lower pole stones based on current AUA and EAU guidelines.

AUA	SWL	RIRS	PCNL
≤10mm	Preferred	Preferred	Discouraged
10-20mm	Discouraged	Allowed	Preferred
>20mm	Discouraged	Allowed	Preferred
**EAU**	**SWL**	**RIRS**	**PCNL**
≤10mm	Preferred	Preferred	Discouraged
10-20mm	Allowed	Allowed	Allowed
>20mm	Discouraged	Discouraged	Preferred

For example, up to half of asymptomatic stones will present in the lower pole, often with a size no larger than 10mm. Natural history data suggests that these are relatively stable entities that infrequently require intervention. At the same time, numerous studies, albeit predominantly retrospective in design, have shown that observation presents minimal risk to patients. Therefore, we favor an initial period of surveillance for asymptomatic stones ≤10mm in the absence of significant risk factors for stone growth, migration, or other complicating factors. Otherwise, consistent with organizational guidelines, treatment is best managed with RIRS or SWL, with preference given to RIRS if unfavorable factors are present. Notably, there is inconsistency in the reporting of patient satisfaction outcomes for these two interventions, with the studies favoring SWL having been performed earlier and perhaps prior to widespread availability of modern ureteroscopic tools.

For stones larger than 20mm, PCNL is overwhelmingly the preferred treatment strategy independent of stone location, with much of the current debate limited to miniaturization of percutaneous tracts and instruments. Of course, RIRS remains an option for patients unfit for the more morbid PCNL. In line with the AUA, we do not infrequently offer RIRS for stones in this size range following adequate patient discussion, including the potential need for procedural staging - particularly in cases involving highly dense stones, complex anatomy, or a stone burden significantly exceeding the 20mm threshold. However, the limits to which we are willing to push ureteroscopy are far more limited in the lower pole, so we generally do not perform RIRS for lower pole stones exceeding 15mm.

Lower pole stones measuring 10-20mm represent a heterogeneous group for which there is greater controversy compared to stones on either end of the size spectrum. This is evident in the discrepancies between organizational recommendations. Unlike their European counterparts, the AUA takes a firmer stance on how such stones should or should not be managed. As previously discussed, the versatility of RIRS allows it to fill a niche for 10-20mm stones in the lower pole. Where SWL experiences a precipitous decline in surgical success for stones >10mm and PCNL confers a significantly higher risk profile relative to surgical benefit in this size range, RIRS serves as a middle ground option. Of course, surgeon skill and experience in treating larger stones with RIRS must not be discounted.

Significant effort has been invested by the urologic community to discern the best course of action in the management of lower pole stones. Indeed, our current understanding of how certain factors, such as stone size or renal anatomy, affect treatment outcomes are the result of over 20 years of collaborative research. While stone-free rates have been predominantly the measure of success, there is growing interest in quality of life and cost effectiveness outcomes as well. To this end, the PUrE randomized controlled trial (ISRCTN 98970319) is an ongoing study from the United Kingdom which seeks to address these research gaps in a direct comparison of SWL, RIRS, and PCNL for lower pole stones (7). Needless to say, the results of this trial are awaited with great interest.

Finally, we must factor our treatment strategies through the lens of surgical innovation. The field of endourology is currently amidst a period of rapid technological advancement, as seen with the growing availability of next generation Holmium laser systems featuring pulse modulation and high power settings as well as the advent of high frequency Thulium fiber laser systems. Single use ureteroscopes also represent a potentially disruptive technology, particularly for the surgical management of lower pole stones. The marked degree of ureteroscopic deflection and torquing required to effectively access the lower pole, as well as potential damage to the working channel from optical fiber use in sharp deflection, can take a toll on the lifespan of reusable scopes with attendant cost considerations. Ultimately, these technologies offer great potential to improve the effectiveness, safety, and efficiency with which we treat lower pole stones of increasing size and complexity. Time will tell if and how they influence our approach to lower pole stones and urinary stones in general.

## CONCLUSIONS

Lower pole stones can pose amplified anatomical considerations that influence surgical success beyond stone size alone. The selected treatment approach should account for attendant risks and benefits of the intervention within the context of patient preferences and outcome expectations.
